# Endonasal interdural pituitary transposition for resection of a posterior clinoid process enchondroma in a patient with Maffucci syndrome

**DOI:** 10.3171/2020.4.FocusVid.19801

**Published:** 2020-04-01

**Authors:** Lei Zhao, Shuo Zhang, Li Gong, Yan Qu, Lijun Heng

**Affiliations:** Departments of 1 Neurosurgery and; 2 Pathology, Tangdu Hospital, Air Force Medical University, Shaanxi, China

**Keywords:** interdural pituitary transposition, posterior clinoid process, Maffucci syndrome, parasellar lesion, video

## Abstract

Maffucci syndrome is an extremely rare disorder characterized by benign enchondromas, skeletal deformities, and cutaneous lesions composed of abnormal blood vessels. Enchondromas rarely arise in the cranial bones. Interdural pituitary transposition is an effective way to gain access to the posterior clinoid, without affecting the function of the pituitary gland. Here, the authors present a case of a posterior clinoid process enchondroma in a patient with Maffucci syndrome. The tumor was resected via an interdural pituitary transposition fashion. Four months postoperatively, the patient’s oculomotor function had recovered to normal and the function of the pituitary gland was preserved intact.

The video can be found here: https://youtu.be/EYgVwVZuC4g.

## Transcript

This video demonstrates endoscopic endonasal interdural pituitary transposition for resection of a posterior clinoid process enchondroma in a patient with Maffucci syndrome.

**0:32** The patient is a 24-year-old female presented with ptosis and oculomotor deficit lasting for 3 weeks. Her left hand presented with multiple hemangiomas, which is one of the critical diagnostic criteria for Maffucci syndrome. Preoperative MRI showed a left upper clivus lesion, mainly involving the posterior clinoid process. Collateral compensation was evaluated preoperatively using balloon occlusion test. The patient showed acceptable tolerance after temporary balloon occlusion of the left internal carotid artery at the petrous segment.

**1:11** The endoscopic endonasal transcavernous approach with pituitary gland transposition was used to resect the lesion. The patient was placed in supine position. Neuronavigation and Doppler monitoring was set and used intraoperatively.

**1:26** The anterior and inferior walls of sphenoidal sinus were widely opened. Subsequently, bony exposure was continued in the sellar and left parasellar regions. The bone covering the sellar floor and the anterior wall of the left cavernous sinus was removed with high-speed drill.

**1:44** The anterior wall of cavernous sinus was opened with a retractable hook knife. Venous bleeding from cavernous sinus could be stopped with hemostasis agent injection followed by cotton pledget pressing. The paraclival carotid artery was further exposed using eggshell technique.

**2:18** After the anterior wall was further opened, the medial or sphenoidal wall of cavernous sinus was retracted to the right side with pituitary gland. The inferior hypophyseal artery was subsequently coagulated and transected to gain access to the posterior clinoid.

**2:43** After successful creating a transcavernous corridor by interdural pituitary transposition, the cortical bone of upper clivus was drilled open. The lesion involving posterior clinoid process was identified and removed in a piecemeal-by-piecemeal fashion. Microsurgical curette was useful for blunt dissection at this stage. The texture of the lesion was not homogenous. Mixture of both bony and cartilage components could be found inside the mass. The magnified view of the endoscope and the bimanual four-hand technique improved the safety of resection. The involved clival and petrous bone should be removed to the maximum extent to prevent recurrence.

**3:52** Some parts of the lesion were with tight adhesion to the surrounding soft tissue. In situ sharp dissection with microscissors should be used to detach the lesion before its removal. The tip of the posterior clinoid process was usually sharp and irregular due to the ligament attachment. It was also necessary to be detached first and removed carefully in order not to injure the cavernous internal carotid artery.

**4:28** The liquid bone matrix in the superior part of the lesion could be removed with gentle suction. The angled instrument was preferred to be used to resect the mass behind the carotid artery with the assistance of counterforce given by the suction tube.

**4:57** The inferior lateral trunk originated from the horizontal segment of the intracavernous internal carotid artery should be protected intact for the preservation and recovery of the cranial nerve function.

**5:16** The final part of the posterior clinoid process tip was dissected from the interclinoid ligament and removed. Extended bony drilling in the clivus was performed to prevent relapse for this young patient. The corner behind the carotid artery was explored to exclude residual tumor remnants.

**6:14** The pan view under endoscope showed gross-total resection of the lesion. The dura graft was placed under the dura mater as inlay. The nasal septal flap was covered as onlay. The BioGlue was evenly applied at last.

**6:36** The pathological examination indicated the lesion as an enchondroma.

**6:43** On postoperative day 1, the patient’s left eye could perform a slight medial movement. Four months postoperatively, the oculomotor function of the left eye had dramatically recovered to normal and the ptosis was completely resolved. MRI, at this stage, suggested the gross-total resection of the tumor without recurrence. Three-dimensional CT skull base reconstruction also confirmed the result and demonstrated the region of postoperative bony defect. No significant change was indicated by the pre- and postoperative endocrinological function comparison.

**7:25** In summary, endoscopic endonasal transcavernous approach with pituitary gland transposition could take advantage of the corridor provided by the cavernous sinus to gain access to the posterior clinoid area safely. Appropriate venous bleeding control with hemostatic agent injection is essential for maintaining a clear surgical field and improving the safety of the operation. Unilateral resection of the inferior hypophyseal artery does not influence the pituitary function.

## Acknowledgments

The technique used in this case was funded by the Annual Novel Technical Project of Tangdu Hospital; Creativity and Development Funding of Tangdu Hospital (2016LCYJ008, 2018LCYJ006); and Young Scientific and Technological Star Project of Shaanxi (2016KJXX-17).
